# Women’s career motivation: social barriers and enablers in Sudan

**DOI:** 10.3389/fpsyg.2023.1153613

**Published:** 2023-08-31

**Authors:** Souad Mohamed, Aida Abbashar, Hala Abushama

**Affiliations:** ^1^Middle East Centre, London School of Economics and Political Science, London, United Kingdom; ^2^The Gender Studies Institute, University of Khartoum, Khartoum, Sudan

**Keywords:** barriers and enablers, women’s leadership, MENA, Sudan, society, culture, motivation, political

## Abstract

**Introduction:**

This study presents an original contribution by examining an often-neglected country in the Middle East and Northeast Africa (MENA), with a specific focus on women’s career research. It identifies challenges that have created barriers for Sudanese women’s career progression, consequently limiting their opportunities for career and leadership growth. To conceptualize understand women’s career motivations on a global and regional scale, the study conducted an in-depth review and analysis of literature, benchmarked similar countries, and incorporated psychological and organizational behavior theories, alongside examples of women’s empowerment cases from the MENA region.

**Methods:**

The study employs a multifaceted approach that involves exploring psychological and organizational theories, drawing insights from self-efficacy, stereotype, and implicit bias theories, as well as MENA empowerment cases. Additionally, an empirical investigation is conducted through an extensive three-round Delphi study involving 75 Sudanese women leaders from diverse sectors. The empirical findings are crucial for understanding obstacles faced by women and the impact of Sudan’s unique social context on their career paths.

**Results:**

The research findings shed light on the complex interplay of factors creating roadblocks for Sudanese women’s career advancement. Sudan’s distinctive social context significantly shapes and influences women’s career motivations in diverse and interconnected ways. Empirical evidence from the Delphi study underscores the broad impact of these roadblocks, highlighting the multiplicity of challenges faced by women in Sudan. This comprehensive analysis not only aids in comprehending workplace obstacles but also provides valuable insights into the diverse experiences and needs of female employees. The findings emphasize the broad impact of these barriers on women, underscoring their varied challenges.

**Discussion:**

The research holds far-reaching implications. By contextually identifying barriers that impede Sudanese women’s career motivations, the study lays a foundation for targeted solutions. This understanding is grounded in historical, theoretical, and policy-making perspectives, enabling informed strategies to support women’s advancement. The study also offers actionable policy recommendations for governments, workplaces, and stakeholders, facilitating women’s career growth through policy reforms and capacity-building initiatives. Furthermore, its significance extends beyond Sudan, acting as a catalyst for developing gender-responsive policies in similar MENA countries and beyond.

## 1. Introduction

Sudanese women have historically been career-driven and ambitious individuals, with many striving to occupy leadership positions within their society. In doing so, they have contested societal barriers responsible for creating the roadblocks for their career motivation in a variety of independent and intersecting ways. Following the fall of Omar al-Bashir in April 2019, the calls for women’s inclusion within all facets of social, economic, and political life have been louder than ever, particularly because the revolution and the transitional period created a space for women to contest societal norms and discrimination. Sudanese women played a critical role in the revolution and it was a turning point for women’s empowerment and leadership (UN Women, 2019). The United Nations report stated that the revolution created a unique platform for women’s activism, leadership, political participation and their contributions have been essential (UNDP, 2020). Thus, the change of leadership and the revolution led by women created a unique opportunity to address gender inequalities and increase women’s inclusion in all aspects of social, economic and political life in Sudan. While the demands for gender parity have been clear, and inclusivity in the career landscape in Sudan has been widely recognized, research on women’s career motivation in Sudan has been limited. Existing studies seem to focus on the challenges that hinder women’s career development and advancement, rather than the factors that encourage women’s inclusion and empowerment. This emphasizes the need for further research to better understand the specific challenges faced by women in Sudan and their career journeys (Fragile State’s Index, 2020; Etang et al., 2021; Gikandi, 2021).

Schock et al. (2019) states that despite being under scrutiny for decades from numerous disciplines, explanations for the gender gap at the highest levels of leadership positions remain unclear. Nevertheless, research in psychology has shed light on the psychological factors that play a key role in shaping women’s leadership experience and explain their low representation in leadership positions. For instance, Bandura (1977) has reported how self-efficacy plays a significant role in women’s motivation, career aspiration and hence leadership experience. Steele and Aronson (1995) state that self-doubts and negative stereotypes about one’s group can directly affect their performance, and Davies et al. (2005) confirmed that stereotype threat influences women’s leadership aspirations and decisions to whether uptake leadership or subordinate roles. Psychological safety (Edmondson, 1999, 2018) refers to one’s belief to express themselves without fear of negative consequences, which is important for understanding how women leaders can feel comfortable to sharing their ideas and making decisions. Maslow’s (1943) hierarchy of needs theory suggests that individuals have different needs, including social, esteem, and self-actualization needs, sheds light on what motivates leaders to succeed. More recently, psychological factors associated with cognitive, emotional and behavioral assets have been confirmed to influence resilience amongst women (Pillay-Naidoo and Nel, 2022). In this regard, the Women and Leadership Theory Think Tank 2015 highlighted the importance that future theorizing needs to be produced from women’s experience (Storberg-Walker and Madsen, 2017). They identified ‘theorizing women leader development’ as one of the key future research priorities. It seems that much of the literature that exists focuses on the barriers and challenges to women leaders (or aspiring leaders), rather than the enabling factors or models for success. Therefore, it is important to theorize women’s career motivation from women’s experiences and their perspectives.

In prior research, the Principal Investigator of this project began to address questions regarding women’s leadership and career motivation through a qualitative research design by carrying out in-depth semi-structured interviews with women leaders across the MENA region 2015–2022 including Nobel Peace Prize winners, Government Officials, Business Leaders, CEOs, Entrepreneurs, Scientists, Executive Deans, and Philanthropists. The study explored their experiences in developing their leadership, attaining their positions, and reflections upon personal strategies in overcoming barriers to their success whilst committed to their quality of work throughout. As a result of the study, it was acknowledged that theorizing successful strategies to overcome barriers from women across the 22 MENA countries means not addressing state-specific barriers where they exist. It was also concluded that it is particularly important to take context into account when conducting and analyzing research to produce women’s empowerment and leadership development recommendations. Finally, it was also discovered that there is limited research available on the career experiences of Sudanese women (Mohamed and Abbashar, 2022).

Although there was an increase in women’s representation in Sudan following the 2018 revolution and the transitional period, gender inequality continues to persist in Sudan and there is still a long way to go to achieve gender equality (Young, 2020). With the MENA region being one of the two world’s least equal regions in the world (Global Gender Gap Report, 2020), it is becoming increasingly vital to understand what is limiting women’s empowerment and professional progression. Central to this is understanding what motivates and demotivates women to pursue careers despite the barriers they are confronted with. The urgency of this study is specifically important in Sudan, where data and research on women’s leadership and career progression is extremely limited (Mohamed and Abbashar, 2022). Sudan is reported to have only 24.5% female participation in the workforce as of 2019, and recently increased to 29.1% in 2020 its highest value in the last 27 years, and 28.7% in 2021 while its lowest value was 23% in 2009 (Indexmundi, 2019; International Labour Organization, 2020). Research on Sudanese women’s empowerment is very limited. Those carried out are mainly by UNWOMEN, one of which focused on South Sudan only and particularly on South Kordofan (UN Women, 2017). Another study focused on supporting the role of women in politics (Chikoore and Hasabo, 2015). Nevertheless, research on women’s career motivation and leadership development in Sudan is an area that is remains overlooked and the voices of Sudanese women and their lived experience have not been sufficiently documented.

An understanding of the factors that impact women’s career motivations in Sudan is critically important, as it can provide organizations and individuals with an understanding of the best practices for supporting women’s career development and supporting gender parity. The failure to do so may impact women’s empowerment, economic and political development, and contribute to a cycle of patriarchal structures that disregard women’s potential and capabilities. In order to confront these barriers, this present research will explore how societal factors impact women’s career motivation, opportunities for leadership and development in Sudan.

This study examines how societal barriers affect women’s career development in Sudan and ultimately, their leadership prospects and attainment. To demonstrate this relationship, this study analyses how women’s career development produces patterns reflective of self-efficacy theory (Bandura, 1977), implicit bias theory (Greenwald and Banaji, 1995) and stereotype threat theory (Steele and Aronson, 1995), in the social, psychological, personal and organizational aspects of their lives. The results of this study have revealed that according to these theories, stereotypes, biases and discrimination manifest in various ways in women’s lives. An understanding of these theories demonstrates how gender-stereotypical views create social roles for women. Furthermore, the stereotypes underlining these roles transcend into various aspects of women’s lives, shaping their career motivation by espousing stereotypical views on women’s work that can be consciously or unconsciously internalized by individuals. These views are also espoused explicitly and implicitly within society and workplaces. Therefore, societal factors prescribe certain characteristics and social roles to women, which reproduce a cycle of bias, sexism and stereotyping that can impact their leadership progression.

The aim of this research is to examine how Sudanese women’s motivation, career opportunities and access are influenced by these implicit and explicit societal factors, including cultural beliefs and gender norms and how these factors interact with personal, psychological and organizational dynamics to shape their career journeys within the context of Sudan’s current political situation.

Specifically, this paper seeks to answer the following questions:

(1) How do societal factors, such as cultural beliefs and gender norms impact Sudanese women’s career motivations and opportunities? (2) How do these factors interact with psychological, personal and organizational dynamics to shape their career journeys? (3) What policy reforms and capacity-building interventions are needed to support Sudanese women’s career advancement and address the barriers and enablers that impact their career aspirations?

By answering these questions, this research provides women with a better understanding into how their career motivations are shaped by various of factors within their society. In addition, it offers recommendations for supporting women’s career advancement in Sudan, while emphasizing the importance of addressing the barriers and enablers that impact their career aspiration.

## 2. Literature review

In order to make a meaningful contribution to the Middle East and North Africa (MENA) studies and conceptualize an understanding of women’s career motivation in Sudan through a comparative lens, this research has consulted a variety of literature on women’s leadership development in the MENA region. It was revealed in this study that while Sudan’s context is distinctive in many ways, the existing conceptualization of women’s career motivations and advancement in other MENA countries can be useful in developing our understanding of how social factors impact women’s career motivation. This is particularly due to the fact that Sudan shares a similar cultural and social context with other countries in the region. In addition, this study has consulted several psychological theories, in order to conceptualize how societal factors can either negatively or positively impact women’s career motivation. This has been done to entrench the importance of recognizing the psychological impact that biases, stereotypes and discrimination have on women’s career motivations in Sudan.

The existing literature on women’s career progression in the Middle East argues that societal factors, such as stereotyping and social norms, can act as barriers and enablers to women’s career motivation and development, particularly when societal expectations shape women’s choices or restrict their access to possibilities (Alqahtani, 2021). This is in line with psychological theories relevant to women’s career advancement. First, the stereotype threat theory (Steele and Aronson, 1995) argues that an individual’s career aspirations and behavior are impacted by personal characteristics and their environment which manifests in the negative stereotype of their group. This emphasizes that women’s career advancement can be shaped by psychological factors perpetuated by an individual’s environment. Patriarchal sociocultural values, and the ways in which they perpetuate gendered ideas of leadership, can limit women’s career choices and development (Patel et al., 2020). For example, a woman’s performance could be affected by the societal stereotyping of women in leadership, and this limits their opportunities in accessing leadership positions or performing well in those positions. In addition, these societal barriers may be impacted by the existence of a male-centered culture, where women’s leadership is not valued and where their contributions and aspirations are limited and overshadowed by a male dominated society (Alqahtani, 2021). In these contexts, society may regard it as shameful for a woman to be in an equal or higher career position as a man. In turn, this often confines women to spaces and roles that align with more feminine characteristics (Guettaoui and Hadja, 2021). Furthermore, even for those in leadership positions, they may not feel confident and underperform in their leadership roles due to stereotype threats. On the other hand, when institutional environments are empowering and supportive of women’s career advancement, studies have noted that organizations tend to perform better (Hameli et al., 2023), because of the inclusion of diverse opinions and perspectives in top leadership positions (*Ibid.*).

Organizational and societal barriers often intersect and create further impediments to women’s career progression in the MENA region. Stereotypical views of female leaders in the Middle East, which associates them with strictly feminine ways of management, implies that their communal or feminine leadership styles are what have lagged them behind men (Tlaiss and Kauser, 2010), and associates them with a token status. For example, a study conducted by the United Nations Industrial Development Organization (UNIDO) in Egypt, Jordan, Lebanon, Palestine and Tunisia, revealed that women leaders in the entrepreneurial sector face discriminatory work environments as a result of stereotypes and preconceptions about their roles and abilities (Laffineur et al., 2018). This discrimination can lead to lack of access to financial resources and professional development and networking opportunities necessary to excel in entrepreneurial organizations. In this organizational culture, women are isolated and discriminated against (Tlaiss and Kauser, 2010), regardless of their progression or leadership status. These organizational cultures can be regarded as unwelcoming, as they negatively impact women’s career progression and motivations. Furthermore, they are grounded in implicit biases.

Implicit bias theory (Greenwald and Banaji, 1995) emphasize how stereotypes and unconscious bias can act as a barrier and impacts women’s career progression and their interactions in senior management circles. In the context of the MENA region this implicit bias may relate to women’s capabilities and leadership styles that affect others’ perceptions, decisions and behaviors towards these women leaders. This may well lead to discriminatory recruitment and promotion practices. Consequently, this leads to women’s underrepresentation in decision-making positions and reinforces stereotypes about their abilities and qualifications. Thus, to address gender equality, it is imperative to recognize and tackle both explicit discriminations and implicit bias in the decision-making process. Implicit bias theory is also linked to stereotype threat theory, as negative stereotypes can negatively impact individual’s self-esteem and performance. Women leaders, particularly in the MENA region may internalize these negative societal stereotypes in relation to their leadership styles and incompetence and consequently doubt their abilities and underperform.

Lastly, personal and societal factors intersect in critical ways that may impede women’s career progression in the MENA region. Cultural expectations for women to prioritize familial responsibilities can influence career motivation. In a study on women’s leadership in higher education in Saudi Arabia, it was revealed that since Saudi women are traditionally expected to only be mothers and wives, that they feel that they must prioritize their familial responsibilities over their professional ones (Alqahtani, 2021). The pressures of a work-family balance, and the cultural and societal expectations that underpin it, disproportionately impact women’s confidence and can play a role in limiting their leadership progression. This links directly to self-efficacy theory, which emphasizes how an individual’s confidence and belief in their capabilities influence their motivation and performance. Stereotypes and Societal expectation in the MENA region may lead women to experience a lack of self-efficacy which may result in lack of motivation and career progression. Thus, it is imperative for organizations to support in developing and maintaining self-efficacy.

Understanding the societal factors that shape women’s career motivations in the MENA region requires addressing the structural and patriarchal norms that that have created these barriers for women. In this area, it is increasingly important to take into consideration the specific socio-historical context of each country in the region, as the societal barriers experienced by women may have varying root causes. Most studies that touch upon societal support for women’s career journeys identify them generally (Hoare and Gell, 2009; The World Bank, 2019). For instance, increasing women’s visibility in the economic sector has been considered a way in which women can distance themselves from social norms that may be confining (Mahmoud, 2005; Hoare and Gell, 2009). In addition, changing men’s attitudes towards women in leadership positions has also been identified as having the potential to motivate women’s career ambitions (Hoare and Gell, 2009). While some discussions on societal enablers exist, these discussions remain somewhat general, and require a more specialized and tailored lens in order to identify specific societal enablers.

The preceding sections have outlined how various social and psychological factors can negatively impact women’s career motivation overlap and influence one another, ultimately defining women’s experiences in complex and multifaceted ways. The same paradigm exists with relation to the societal factors supporting and motivating women to strive for careers. Specifically, career motivation can be influenced by societal and legislative factors. For instance, The World Bank (2019) notes that supporting women’s mobility and their acquisition of technical skills can play a role in supporting career progression. In addition, supporting women’s access to education (Kemp, 2012) has also been noted as an enabler for women’s career advancement. These enablers can all play a role in addressing hesitancy or fear some women experience because of limited access to professional, educational and practical career support (Alzougool et al., 2021). Supporting women in gaining an education and acquiring leadership skills depends heavily on positive attitudes towards women’s participation.

### 2.1. Sudanese women and career motivation

Historically and more recently (Osman, 2014; UNDP, 2020; UNICEF, 2021), Sudanese women have confronted a variety of social roadblocks that have impacted their career motivations. Within the context of the current political situation, it is growing increasingly important to clearly conceptualise women’s career experiences in Sudan in order to better support their ambitions, motivations, and leadership developments. In addition, it is critical to note that the societal norms, stereotypes and biases experienced by women in the career journeys in Sudan have been structurally constructed by previous governments in a variety of ways throughout Sudan’s post-colonial history (Osman, 2014).

During the post-independence period, Sudan made notable progress in women’s participation. In post-independence Sudan, many women gained education and became doctors, engineers, nurses and teachers (Osman, 2014). In the political arena, the women’s movement was incredibly active, and women gained the right to vote and stand for leadership positions within the government (Osman, 2014). Even during times of political change and turmoil, the calls for Sudanese women’s participation in the public sphere have remained evident, with women leading protests and demanding their rightful inclusion in society. Nonetheless, shifting socio-cultural beliefs and social norms continue to meet resistance from individuals and groups whose interest is the maintenance of the status quo (UNICEF, 2021). A long history of government sanctioned legislations and policies have limited women’s roles and created roadblocks for their career progression. Over the 30 years laws were applied which limited women’s access to the public sphere, as it required them to seek permission from male guardians to work outside of their homes (Osman, 2014). For example, the 1992 Public Order Act (Gikandi, 2021) limited women’s access to work outside the home. The 1997 Labour Code also reinforced societal norms and customs, which limited Sudanese women’s potential, particularly economic potential (Etang et al., 2021). In doing so, these laws simultaneously strengthened male presence in the workplace while creating exclusionary environments for women.

Other policies and legislations limited women’s access to training and further education, which in turn impacted their opportunity to access the job market and progress in their careers (Osman, 2014). In Sudan, women enjoy less than a third of the rights that men do in relation to economic opportunities according to the World Bank’s ‘Women, Business and Law 2020’ report, which scores this against eight different measures including women’s mobility, marriage, parenthood, pay, entrepreneurship, assets and pensions – all which Sudan has not scored improvement on over the past decade (Etang et al., 2021). While the 1992 Public Order Law was removed by the transitional government in 2020 (Fragile State’s Index, 2020), its legacy remains embedded in Sudanese society.

In addition to legislations and policies, patriarchal mentality has created hurdles for women’s career motivations and ambitions (Tonnnessen and Al-Naggar, 2020). At times when women’s representation has been advocated for and their role in the development of the country has been highlighted, it has been argued that they were only permitted to perform the roles designed for them by the ruling patriarch (Halim, 2019). The UNDP (2020) report on women’s participation in politics in Sudan infers that 32% of the surveyed sample believe that discriminatory traditional and cultural norms are a key barrier to women’s career advancement. At the basis of this are societal gender norms, that have prescribed Sudanese women to service-oriented, home-focused responsibilities while placing men in positions of decision-making (Gikandi, 2021) and leadership. In doing so, society has disenfranchised Sudanese women, depriving them of networks necessary for recruitment and career advancement. Coupled with legislative limitations, the projection of societal norms creates structural challenges that impact Sudanese women’s motivation to seek work outside of the home, particularly because they are likely to be faced with embedded discriminatory beliefs.

## 3. Methodology

### 3.1. Participants

In selecting the participants of this study, purposive sampling and key informants approach were combined as well-established methods to ensure validity and reliability of the data collected. The sample in this study consisted of 75 participants, ages ranged from 25 to 80 years old, they held a variety of different job titles, including, but not limited to, CEOs, heads of organizations, founders, researchers, and professors.

The participants recruited using purposive sampling (Palinkas et al., 2015), as representatives of women leaders working in Sudan: (1) university academics in the areas of gender, diversity, leadership development and women studies, (2) non-governmental organizations (NGOs) and international non-governmental organizations (INGOs), governmental bodies, and policymakers (3) a mix of business practitioners, and (4) Sudanese women leaders. These groups were selected to provide a comprehensive understanding the barriers and enablers faced by women and to ensure the sample is representative of Sudanese women across sectors.

Participants were then allocated to each of these groups according to their field of expertise using the key informant approach, which has been used extensively and successfully in numerous divisions of social science studies (Laffrey and Page, 1989; Perkins, 1992; Marshall, 1996). Key informant approach was specifically selected to attain insights from Sudanese women who have the relevant expertise and are able to provide valuable account of their experiences and needs of women leaders particularly in the context of Sudan and can help to further inform the research question. The participants met the five criteria of eligibility as key knowledge holders (Marshall, 1996) ensuring that the study results are robust and informative.

The key eligibility criteria for identifying participants were:

1. Expertise: women leaders who have extensive knowledge and experience in their respective fields,
2. Leadership: The key informants should have held leadership positions in their workplace or community, demonstrating their ability to effectively navigate and advance their careers while overcoming obstacles and challenges.
3. Diversity: The key informants should come from diverse backgrounds, including different industries, educational backgrounds, and socioeconomic statuses, in order to provide a comprehensive and well-rounded perspective.
4. Experience: The key informants should have at least 3–5 years of experience working in their respective fields, demonstrating their understanding of the industry’s challenges and opportunities.
5. Willingness to participate: The key informants should be willing to participate in a three-round Delphi study and be available and responsive during the study period.

### 3.2. Method and data collection

#### 3.2.1. Delphi technique

The origins of the empirical data in this project came from a three-round Delphi study questionnaire. The Delphi technique was used to gain consensus from a panel of experts using a series of questionnaires and to provide a provision of feedback to the participants (Linstone and Turoff, 2002). This technique is predominantly used in the social sciences for understanding underexplored areas with limited theorizing (Chan, 2022). The Delphi technique is a structured and iterative process which is very beneficial in developing consensus among participants and using feedback to revise, refine, and validate data collection instruments. In doing so, ensuring the questions are clear, concise, and relevant to the research question appropriate for the target group. This approach was particularly selected to enable us explore women leadership development in Sudan, prioritize the key barriers to women advancement in their career, and identify enablers for overcoming them.

The Delphi technique was utilized to create open questions over the course of the three rounds. Participants were informed that the study aimed to develop an understanding of the barriers and enablers they have encountered in their career journeys and were asked to complete three anonymized questionnaires as part of the study. The purpose of this three-phase process was to validate the data and receive controlled and reliable opinion feedback (Chan, 2022). The questions asked were open-ended, in order to allow participants to expand on their experiences with detailed answers. The first round of the Delphi incorporated questions based on a thorough literature review and existing knowledge on Sudanese women’s leadership development. Following the coding and analysis process, round 2 of the Delphi questionnaire was developed from the gaps identified in the previous round. The open-ended questions held two components: identification and validation questions. Following the coding and analysis of Round 2, Round 3 questionnaire was developed with a focus on policy reform in accordance to what the data has identified thus far.

While the questionnaire was anonymized, participants were asked for the following four attributes: position titles, years of experience, sector, and organization size. This was done to support exploratory thinking in the analysis and to identify any sector-specific trends.

#### 3.2.2. Qualitative coding and data analysis

Data was analyzed using content analysis. Content analysis was selected to analyze patterns found in the open-ended questions and to allow the research team to identify and compare the barriers and enablers experienced by women during the career journeys. This approach allows for the coding of raw text into selected classification schemes and has been frequently used by qualitative researchers to derive meanings from texts concerning career motivations (e.g., Kondracki et al., 2002; Cheriyan et al., 2021). The content analysis for this study was conducted by the researchers using NVIVO on a secure and anonymized basis. NVIVO was specifically selected for this project due to its ability to analyze and organize the text, identify relevant themes and categorize the respondents based on the limited profile information.

The coding process for the participants’ data involved a hierarchical structure for analysis, which aimed to classify each respondent according to their specific attributes. Each response was coded via cases and codes, with the case option allowing the research team to save each questionnaire received by the respondents under their individual cases. This approach facilitated tracking trends in responses from individual participants and identifying common themes based on each participant’s case classification. To protect the privacy of the participants, the responses were anonymized. Each participant was assigned a unique identifier (case), which was used to match their responses across different questions. This ensured accurate data analysis while maintaining participant anonymity. Moreover, case classifications allowed the research team to filter and visualize participant responses based on their specific attributes, such as position, years of experience, sector, and organization’s size. Once each participant was classified under a specific case, their questionnaire was coded to establish common themes related to women’s career motivations in Sudan. These themes were then merged and organized to provide an understanding of women’s career motivations, ambitions, and advancement in Sudan.

#### 3.2.3. Validity and reliability

The Delphi technique was used to create a consensus among the experts on the research protocol and to refine the survey instrument used to collect data from the participants. The validity and reliability of the data collected from the participants were ensured by using a consistent research design and a representative sample of women leaders with specialized knowledge and experience. Data collection stopped at round three of the Delphi questionnaire when saturation was reached. Content analysis was carried out in each round to identify common themes across sectors, ensuring the generalizability of the findings.

## 4. Results

### 4.1. Barriers to women’s career motivation

Three rounds of the Delphi study questionnaire revealed, across sectors, that stereotypes and negative perceptions about women leaders and their abilities espoused within Sudanese society have impacted women’s career motivations. Specifically, patriarchal culture and mentality, grounded in cultural and social beliefs that women do not need to have career motivations, has led to Sudanese women fearing discrimination in hiring. For women within the workplace, this fear extends to discrimination in promotion, pay and involvement in tasks that could further their career ambitions. It was revealed that this is further heightened by young age, which participants have noted can also impact their confidence in pursuing careers and striving for leadership positions, particularly because there is a fear that employers will not take young women seriously.

Another cross-sectoral societal factor impacting women’s career motivation is resource disenfranchisement. Study participants noted that social standing can impact the access women have to social, economic and educational resources. This is underpinned by specific societal beliefs regarding the appropriate spaces in which women should operate (Guettaoui and Hadja, 2021), and has disempowered women’s career motivation in Sudan. It was noted in the study that possessing a specific social capital can allow women to access professional development opportunities in Sudan. Namely, coming from a prominent family in Sudan or having access to international networks were cited as factors that ease women’s career journeys. In the same breadth, it was also noted by participants that women from marginalized backgrounds in Sudan may have less access to economic and educational opportunities.

### 4.2. Enablers to women’s career motivation

This study also revealed that to support women’s career motivations, governmental and workplace policy and program reform is critical. First, participants agreed that policies should be instilled that prohibit gender-based discrimination within hiring and promoting practices. In addition, it was noted that across sectors, there is a need to introduce anti-discrimination and women’s empowerment modules at educational and workplace levels. Early introduction to the importance of anti-discrimination and women’s empowerment within the Sudanese education system can support stopping cycles of stereotyping, implicit bias and negative perceptions about women from an early age. In addition, this training should continue, with the support of federal and local governments, at workplace levels. Specifically, participants across sectors have argued that hiring managers should undergo anti-bias training, and staff and senior management across all genders should undergo training on the importance of gender inclusivity in the workplace. Finally, participants have underlined the importance for stakeholders focused on women’s empowerment in Sudan to confront their own implicit biases and stereotypes when devising ways to support women’s career motivations in Sudan. As discussed, the societal conditions that have served as barriers to women’s career advancement are structural and entrenched. As a result, participants noted that there is a desire for safe, accessible and anti-discriminatory opportunities to emerge in education and in the workplace. These opportunities should possess the overarching aim of supporting women in realizing their career ambitions and motivations without fear of societal barriers.

## 5. Discussion

In order to understand how women’s career motivations have been shaped by societal factors and their psychological impacts, it is first critical to understand how Sudan’s current political situation has shaped women’s experiences. Through a contextual lens, it becomes clear that women in Sudan have experienced distinctive challenges and opportunities since the revolution and preceding political changes in the country. This context is particularly important to recognize, as Sudan is in a period of political transformation that can potentially play a role in motivating women to follow their career dreams in the future. Ultimately, the challenges in the country have accentuated patterns of social exclusion and contributed to patterns of stereotyping and biases against women. In view of that, shifting the social norms and socio-cultural beliefs in Sudan continue to meet resistance particularly by those whose interest is maintaining the status quo (UNICEF, 2021). Women’s career ambitions bear the brunt of this.

Political challenges in Sudan have led to a turbulent economic situation which in turn has accentuated existing social exclusion. Women are particularly disenfranchised by this, as they have been historically marginalized and men are usually considered the breadwinners of their families. Osman (2014) argues that some policies and legislations limited women’s access to training and further education, which in turn impacted their opportunity to access the job market and progress in their career. As such, men have more access to economic opportunities. In view of the widening of the gender gap in the labor force, women are more susceptible to being impacted by the cost-of-living crisis (Rivi, 2022).

This dynamic disproportionately impacts women living outside of Khartoum, who have experienced economic disenfranchisement for decades. This is particularly the case in regions impacted by conflict. Speaking on loss and disenfranchisement, a female activist and unionist noted, ‘many times when I have experienced difficulties, especially during the war that broke out in the Nuba Mountains in 2011, I had lost everything. My house, my work, and my husband fled and became a refugee in South Sudan. My family helped me to start all over again.’ Improving the current economic and conflict situations in Sudan and ensuring that policies support women’s access to economic opportunities would enable women to have access to the resources necessary to support their career motivations and development.

There has also been a lack of focus on marginalized women in Sudan, which has led to the polarization and exclusion throughout the country and has not led to sustainable change in the area of all women’s rights and concerns. There’s an understandable disappointment with this, particularly due to fact that there was an expectation that the revolution would open more opportunities for women to chase their professional ambitions. However, the revolution can still be regarded as an example of women’s capabilities and motivations. A woman leader, who played a central role in mobilizing efforts during the revolution, noted that women’s wide participation (70%) in the 2019 revolution (Young, 2020) has made more people aware of women’s role in political transformation and it has given us more opportunities to participate. Although women’s participation is not to the level desired, women have organized to a high degree and have been pushing for their voice to be heard.

Ultimately, the impact of societal factors on women’s career motivation in Sudan is grouped in the following manner: (a) societal factors influencing women’s career motivation, (b) societal and personal factors that in conjunction influence women’s career motivation, and (c) societal factors and organizational factors that together influence women’s career motivation. In addition, throughout these groupings, the role that implicit bias theory, self-efficiency theory and stereotype threat theory play in women’s career motivation and development become evident in intersecting and independent ways.

### 5.1. Societal factors, personal factors, and women’s career motivation in Sudan

As discussed, Sudan’s societal norms and conditions are engrained in legal and structural mechanisms, which operate alongside culture and tradition. As such, there is the existence of a male-cantered culture, that has the power to shape women’s choices and access in the careers (Patel et al., 2020; Hameli et al., 2023). This male-cantered culture has the capacity to shape women’s choices and access (Alqahtani, 2021) because stereotypes and negative perceptions about women’s career advancement overshadow their potential and personal motivation. Ultimately, these create barriers to women’s careers and can have demotivating impacts on the individual as it signals the potential for discrimination during their career journey. This study has revealed that stereotypes and negative perceptions about women’s career advancement in Sudan creates a cycle of discouragement, whereby women bear the brunt of societal expectations and norms in personal and professional facets of life.

On a personal and psychological level, an individual’s community and family environment can shape their career motivation in various ways. Having supportive community or family networks can be encouraging for women’s confidence and ambitions. Participants noted that being from households where they were encouraged to be leaders and having families that supported their education and careers has opened many opportunities. Some women even argued that their families engrained career values in them from a young age, while simultaneously providing them with financial support to pursue their education. When speaking about her family, a former Director of a national ministry said “they were always supportive of my ambitions, and much was attributed to their openness and ease of thinking. Growing up, my voice was always heard as a family member, which built my self-confidence and ability to lead and express my thoughts publicly without reservation. Growing older, I was still counting on my family to support me with my baby while I was away for work and traveling.” A prominent Sudanese activist reflected similar sentiments: “I came from a family who support girl’s education and encouraged women to speak out. For example, my father encouraged me to stand before a crowd and talk when I was 14 years old. My family also pushed me gently to participate in school outdoors activities which later influenced my leadership skills a great deal.” Evidently, supportive networks can play an important role in women’s career motivation. In situations where women receive no external support for their career journeys, their leadership development may be impeded (Serfaz and Mehta, 2019). Drawing on self-efficacy theory (Bandura, 1977), there is a clear correlation between possessing a positive support network and psychological factors such as self-confidence, self-esteem and an individual’s belief in their own success. The presence of positive support network improves these psychological factors and enhances self-efficacy. As these examples have demonstrated, these support networks affected how the women in these studied viewed themselves and felt about their career journeys. As such, they felt motivated to pursue their career goals, despite societal norms and challenges.

While supportive networks can play an important role in supporting women’s career motivations despite societal norms and conditions, in Sudan’s case, this support and access to opportunities is closely tied to financial stability. For most participants in this study, being from a financially stable household opened educational opportunities to study at high-ranking universities in Sudan and abroad. As a result, women can access opportunities that have the potential to inspire and direct their career journeys. This level of access can play a vital role in addressing hesitancy or fear some women experience because of limited access to professional, educational and practical career support (Alzougool et al., 2021). A Manager at a private sector company noted, “my education is an important factor. The opportunity to access higher education enhanced (my) upskilling opportunities in leadership and employment. (It) also effected (my) career and salaries.” Evidently, as well as skills development, this participant believed that access to higher education made them more marketable. As Kemp (2012) notes, supporting women’s access to education can enable their leadership journeys. In addition, education can address the hesitancy or fear some women may have throughout their leadership journeys, by equipping them with the tools necessary to succeed in their fields (Alzougool et al., 2021). Having access to these types of opportunities can be motivating because they open various avenues for career advancement and life choices.

Closely tied to education is financial support, which was another aspect identified by participants as something that can have motivating effects. Participants noted that financial support from their families from a young age played a major role in allowing them to pursue their education, travel and undertake extra-curricular activities, all of which have opened the door to leadership opportunities and skills development. As a start-up co-founder reflected: “My economic background has made certain opportunities available to be in comparison to others.” In addition, participants noted that financial support has allowed them to pursue roles that are low paying, which is a consequence of Sudan’s poor salary environment. A former Ministerial employee noted that “I continue work in the research center although salaries and incentives are very low, mainly because I have no economic burdens in my home, my husband is responsible from all expenses.” Evidently, across sectors, financial support has played an important role in providing women with access to education and skills development necessary to fulfil their career ambitions and motivations. This access is dependent on positive attitudes towards women fulfilling their career ambitions. However, this access is a privilege. Sudan’s current political, economic and conflict situation has meant that resource disenfranchisement is prevalent across the country at social, economic and educational levels. As the literature notes, women’s lack of access to social, economic and educational resources (Guettaoui and Hadja, 2021), has served as a barrier to women’s career advancement. In Sudan, this disenfranchisement disproportionately impacts women living outside of Khartoum and women from marginalized backgrounds. Hence, it is evident that lack of access to educational and financial resources can impact an individual’s self-esteem and self-efficacy, resulting in yet another roadblock to women’s career aspirations and advancement.

Another societal factor that has shaped women’s career motivations in Sudan are household responsibilities. Along with their career ambitions, many women have familial ambitions. Societally, women are often regarded as responsible in retaining a balance between work and family ambitions and commitments (Evans and Maley, 2020). While it is important to note that this responsibility is often overemphasized as a result of stereotypes about gender roles, it is very much a responsibility that many women confront at some point in their career journeys. The struggle to balance work and family commitments, especially in contexts where there is no external support, may impede a woman’s career development (Serfaz and Mehta, 2019). As Alqahtani (2021) notes, there is a traditional expectation in the MENA region for women to prioritize familial responsibilities over their professional ones. The pressures of a work-family balance, and the cultural and societal expectations that underpin it, disproportionately impact women and can play a role in limiting their career motivations. In this study, working mothers noted that this struggle limits the amount of time they can spend focused on their career, and that they feel they need to compromise their career ambitions in order to put child-care obligations first.

As a working mother, said an advisor to a government Ministry, “it was hard to balance work and life commitments.” A business manager also pointed out that “the long hours that are associated with higher level of responsibilities always perceived as unaccepted from the society and communities we lived in in Sudan. This happened only for women while this is considered normal for men who are holding the same responsibilities at work.” As these quotes demonstrate, women in Sudan may find themselves at a crossroads between prioritizing their work or family ambitions. Ultimately, as pointed out by several respondents, there is an underlying social pressure in Sudan about the importance of marriage and children. A Manager at an international organization argues that ‘social pressure around the age of marriage and the need to form family and get kids at early ages’ can be a large constraint. Evidently, women in Sudan are increasingly conscious of the household roles prescribed to them. Arising from this consciousness, is the risk of being labelled an absent mother or wife, due to pursuing your career ambitions. This labelling exists throughout the MENA region because women’s responsibilities at home are emphasized by society (Khurma et al., 2020). As Steele and Aronson (1995) argue, individuals go through self-doubts when they are aware and conscious about negative attributes associated with their group. In this case, there are negative stereotypes associated with being an absent mother or wife within Sudanese society. Ultimately, this social pressure can make it difficult for women to balance their personal and work obligations and develop their career motivations.

### 5.2. Organizational factors, societal norms, and women’s career motivation in Sudan

Alongside personal factors, organizational factors also intersect with societal norms and conditions and influence women’s career motivation in Sudan. Organizational barriers to women’s careers are intrinsically linked to organizational culture and practices. Within these culture and practices, stereotypes and biases may be projected onto women in the workplace. These stereotypes and biases can influence women’s career motivations because they can create either inclusive or discriminatory environments. Discriminatory work environments are often underlined by biases projected at several levels of an organization – whether that is at C-Suite level, amongst staff, or within hiring divisions. Institutional conditions have a major role to play in women’s inclusion and empowerment (Hameli et al., 2023). These conditions can either negatively or positively impact women’s self-esteem, confidence, ambitions, and career development. A woman leader and Director of an organization noted, “I do lack confidence in a lot of aspects, and I think my lack of confidence stems from my lack of experience in the beginning and moving forward, as I was able to garner more experience I am still not necessarily confident in my abilities and a huge part of that is related to how my family does not value what it is that I am doing.” These insecurities are driven by a variety of factors, including uncertainty of competencies, which are perpetuated by negative stereotypes associated with ambitious women in Sudan, as well as a lack of access to information and knowledge that could be useful for their career journeys, namely skills development programs or policies in support of women’s empowerment. In addition, male domination in specific sectors has been responsible for creating a culture of intimidation, which can impact women’s confidence, and ultimately, career progression (Patel et al., 2020).

Furthermore, implicit biases exist at policymaking levels in workplaces in Sudan. There is a lack of gender sensitive policies that support women’s employment and support them when they are hired. This includes a lack of clarity and existence of code of conduct policies and a lack of gender sensitive policies that address female-specific needs such as childcare, maternity leave, female health and bathroom supplies and facilities, and flexible working arrangements. On maternity leave and childcare, a General Manager from the private sector said: “One of the policies I would really like to see applied in my organization is paid long maternity leave for parents. This policy will allow women to pursue their careers while still having the time to have and raise her children.” Similarly, a brand manager said “I mentioned before that my workplace is meeting a lot of our needs as women, thankfully. But I really hope we can get more attention on childcare. We have mothers who struggle to provide care for their children while working. This consumes a lot of their pay-check and time.” Gender norms that are placed on working women underline the expectation that women must attend household responsibilities over work-related ones (Khurma et al., 2020). On the other hand, studies demonstrate that fathers are not expected to make sacrifices at work for the sake of their household responsibilities (*Ibid*). On flexible working policies, a manager in the private sector said these policies are important, especially in Sudan’s current situation “where security and economic challenges have affected our daily routines (flexible hours, working from home polices for example).” It was believed by participants that these type of gender sensitive policies are essential for confronting patriarchal mindsets. Studies demonstrate that adopting policies such as childcare support and flexible working hours can often alleviate the double burden that working mothers (*Ibid*). Ultimately, through instilling these gender-sensitive policies, organizations will be able to engage more women at leadership levels.

Implicit biases may also hinder the access women have to professional development and mentoring opportunities. Male-normed organizational culture and networks can underplay the importance of women’s access to professional development and mentoring opportunities. Furthermore, male-centeredness platforms the experiences of male employees as the norm, while stigmatizing the experiences of female employees (Kim, 2020). This can lead to a lack of recognition of women’s experiences, positive or negative, within the workplace. Most literature on women’s career advancement recognizes that mentorship, organizational networks and interpersonal relationships are considered to play a central role in the advancement and selection of managers in the workplace. With regards to mentoring, research has demonstrated that women in the MENA region often have limited access to mentors, which can negatively impact their career development (Tlaiss and Kauser, 2010). Mentoring is widely considered to be a central way in which managers advance in organizations (Tlaiss and Kauser, 2010). Therefore, the absence of mentors or mentoring programs for women in the MENA region can be considered an organizational concern, as it limits their access to career opportunities. In addition to mentoring, organizational networks and interpersonal relationships play a critical role in access to managerial positions. This is specifically true in the MENA region, where the selection process is considered highly subjective and heavily based on contacts and family name (Hutchings and Weir, 2006). In Abalkhail’s (2019) study on women’s leadership in the United Arab Emirates, the author noted that professional networks are heavily male dominated. Limited access to organizational networks and interpersonal relations implies that MENA women are more likely to struggle to access professional support, organizational information, and career planning (Tlaiss and Kauser, 2010).

### 5.3. Mentorship training and women’s career motivation

Mentorship training has been proven to play a crucial role in women’s leadership development (Alzougool et al., 2021). In Sudan, the mentorship training available to women is often unstructured and insufficient in addressing the concerns of women leaders. “It was hard finding the right mentorship and capacity building resources to build my personal capacity and leadership,” noted a female founder of a development organization. There is also a lack of structured mentorship available to women leaders. “The mentorship that does exist usually happens by accident or periodically and is usually not from (within) the sector, so this makes it hard to understand how to progress,” pointed out an international non-governmental organization (INGO) Principal. These types of trends have led to a lack of skills development and has forced women who have the financial means to look outside of Sudan for developmental initiatives and mentorship, as opposed to utilizing local resources. These outside resources have also been noted as insufficient, as they do not consider Sudan-specific contexts. To address this barrier, organizations can fund or partake in their own mentorship, networking and professional opportunities for women in the companies (*Ibid*). These programs will be especially useful if they include professional leadership coaching, networking, soft skills training and situational analysis training. A general manager from the business sector noted that “leadership programs that helped me to hone my soft skills (understanding people, leading others, adapt to change, strategic thinking, improve creativity, etc…).” Whether these programs were held internationally or by the organization itself was unclear, and it was evident that those working in international or regional organizations or private sector organizations with an international or regional organizations had more access to these programs than those operating in local contexts. Ultimately, without access to these opportunities, women may feel demotivated to advance their careers or enter the job market. Mentoring and professional development opportunities are critical, particularly because they can help women realize their potential and ambitions. In addition, through these means, women leaders in Sudan will have the opportunity to create strong networks between each other, where they can support, advocate and learn from one another (Patel et al., 2020).

### 5.4. The importance of managerial support

Finally, implicit biases espoused by hiring managers and senior management is another factor that can impact women’s career motivation. In line with gender-stereotypical views, gendered social roles emphasize that women are more communal, while men are more agentic (Alqahtani, 2021). These types of stereotypes transcend to the workspace, with women being associated with jobs that most likely reflect their social roles, such as jobs that require helpfulness and collaboration. These perceptions create societal barriers in two ways. Firstly, they reemphasize stereotypes about men and women, which classify men as being more likely to be efficient leaders. In addition, these stereotypes contribute to prejudice and bias, where women may be perceived as less suitable leaders because of their stereotypical gender roles (Evans and Maley, 2020), and are therefore less likely to be promoted to leadership positions. When hiring managers and senior management preface promotion, hiring, and engagement practices on their own biases towards women, it becomes increasingly difficult for women to feel motivated in the workplace. In fact, these implicit biases can have polarizing and exclusionary effects. They can also impact women’s self-esteem in a negative manner. Two managers at INGOs emphasized this by sharing their experiences. “As a woman and an activist, who is working to establish a public feminist institution in polarized and patriarchal setups, my leadership is resented among both women and men who are part of public spaces,” noted one participant. The other participant highlighted her experience by saying: “As a junior woman in my field, my opinions were often dismissed. The specific environment of my recent job, my communication was very limited, since my supervisor did not allow much of it.” Participants argued that having unsupportive senior management has led to polarization within their organizations. This polarizing work culture also has meant that some women feel unmotivated to develop in their careers.

On the other hand, the participants that had supportive senior management argued that their managers played a central role in their career progression an international development expert said “I have had incredibly supportive female managers who have modelled me and allowed me the space to make mistakes, pitch to senior leadership and believed in ideas/initiatives and challenged me to make them better. I have also had male leaders who have taken an interest in seeing me grow professionally, made me understand how to present my ideas better and not been threatened by my gender.” Implicit biases can be debilitating and exclusionary. On the other hand, having supportive senior managers can be a motivating factor that opens opportunities from women’s career advancement.

## 6. Policy implications and a way forward

Evidently, Sudanese women’s career motivation, opportunities and access have been shaped by a variety of psychological, personal and organizational factors. These factors are grounded heavily in societal norms and expectations on women’s role in society, as well as their capabilities. There are several policy implications that may be drawn from this study. First, this research provides opportunities for the Government of Sudan (GoS), organizations and companies in Sudan, and relevant international and national organizations to think about the impact of societal barriers to women’s career motivations and journeys. The women who participated in this study noted that they experienced conflict in striving for their career ambitions while also fulfilling their societal roles. Also, they noted that the current political situation has created insecurity and safety concerns, which can sometimes even impact their career motivations. In addition, the participants argued that biases and stereotypes on women’s capabilities have arisen at various points in their career journeys, including within the workplace. Finally, women want to feel motivated in working towards their career journeys, regardless of what is considered socially appropriate. In particular, there was a desire to have equal access to career development opportunities, regardless of an individual’s gender or socio-economic background.

### 6.1. Security sector reform

There are several policy implications for stakeholders to consider considering these findings. In order to do so effectively, Sudan’s political situation should be considered. Security concerns, particularly post October 2021 coup, have heightened throughout Sudan. These concerns disproportionately impact women, who live in fear of experiencing various forms of violence, even when carrying out day-to-day tasks such as going home from work. Furthermore, the lack of security in Sudan have made some participants question whether it is safe or worth it to have career ambitions within the current political climate. A manager from an international organization noted that “the political instability and associated security issues are hindering women participation in the public sphere as general.” More specifically, these security concerns can impact how safe women feel when going to work. “All the stress and uncertainty make work harder for us as women, when you have basic needs like the security it’s even harder feeling responsible for the safety of others during work hours, I know this sounds silly but we as women could worry more about if our team member made it home safe,” said a manager from the private sector. Questions of safety can have detrimental effects on career motivations and these effects disproportionally impact women. Policy interventions from the government to protect women and improve the security situation can mitigate these risks and fears; “As grown-up women now I still feel it’s effect on us and I still fear arresting (calling it Kasha here) when I see young girls in the streets,” said a head of department at a federal ministry.” In addition, instilling grievance mechanisms so that women can easily access reliable methods of reporting is also critical in renewing women’s trust in the police and government. Finally, policies that support women from abuse by the police should be instilled. Particularly because the public order law has been removed, it should be replaced by a framework that supports women’s safety and security.

### 6.2. Organizational policy reform

From an organizational perspective, anti-bias and inclusion practices can encourage women to feel motivated in their career journeys and feel included in tasks in the workplace. Sudanese women across sectors would benefit from supportive and gender-sensitive managers. Anti-gender bias policies for hiring and promoting women within workplaces, adequate checks and balances systems for addressing issues of alleged discrimination, and implicit bias trainings for senior managers are all factors that can address gaps in support and inclusivity. Gender-sensitive policies in the workplace can also play a role in supporting women’s career motivation. As this paper has demonstrated, there is a societal expectation for women to prioritize marriage and children over their career ambitions. In order to confront this and motivate mothers to pursue careers along with having families, workplaces can instill gender-sensitive work policies. Flexible working policies, maternity and paternity leaves, and in-house child-care facilities are all factors that organizations in Sudan can enshrine to support women’s career motivations and advancement. It is important that senior management and staff understand the importance of supporting women with familial obligations. Supporting working mothers and those with familial obligations can allow workplaces to reap the benefit of a wider workforce. To support the understanding of this rational, workplace staff can undergo gender-sensitive modules offered by gender experts, which will shed light on how gender-sensitive working conditions can support women’s work and lead to more productivity within the organization.

Finally, there are ways in which organizations can help lessen the psychological impact of imposter syndrome (Clance and Imes, 1978) and low self-esteem felt by several women at various points in their career journeys. For example, a Chief Officer in the private sector said: “The thing is that when I had the coach, I was discussing with her life concerns of having to prove myself, and then she really rightfully made me realize that these concerns were only in my head. So I was the one who anchored myself down by thinking people were judging me or people were thinking I need to work more. I did not need to prove to myself I was doing my job, and that’s it.” Misconceptions and societal biases towards women’s capabilities to succeed in their careers is a factor that impacts women’s motivation to pursue work. To address this, structured mentorship programs can play an important role in supporting women’s confidence and addressing a variety of insecurities. While mentorship programs do exist in Sudan, many of them are informal. Structured programs starting from education and from within the workplace can play a consistent role in shaping women’s confidence and providing them with guidance in their career journey.

A previous government Minister noted, “I was exposed to different people with different backgrounds and contexts. (This) sharpened my skills in leading a team with differences towards a common goal and (developed my) ability to understand this difference and synthesize a self-paced vision collectively. This skill also enabled me to be more patient and careful.” Similarly, a Chairperson of a Union said, “I joined some national organizations and associations which are specialized in culture, heritage and women’s issues. That was so useful (for my) self-confidence and (in helping me) become closer to my people. In addition, I recognized in (a) very specific way what (the) Sudanese women’s agenda (is) and how to work in group to make it possible and true.” Evidently, providing opportunities for women to develop their confidence can motivate them to pursue their work ambitions, while also giving them the opportunity to work with likeminded individuals with the common goal of supporting women’s empowerment and inclusion.

### 6.3. Career development opportunities

While there are several avenues for policymaking and programs to intervene and support women’s career ambitions and advancement, access to career advancement opportunities may not be as easily available to all women in Sudan. An individual’s socio-economic standing can play a role in determining what opportunities are afforded to them, specifically in the realm of education. This study demonstrated that education has played a role in motivating women to pursue their careers. However, women in Sudan who are affected by poverty and conflict may have less access to educational opportunities. The GoS has a role to play in increasing the educational opportunities available to marginalized women. By allocating funding to support women across Sudan in accessing education, the GoS will allow more women to develop their knowledge, skills, and networks – all of which are factors that can motivate women to pursue careers. National-level social protection programs can play an important role here too, particularly by providing financial support and scholarships to female students to attend courses, technical vocational and educational training (TVET), and national and international universities.

### 6.4. The Government of Sudan’s (GoS) policy-making role

The GoS has a role to play in addressing implicit biases and discrimination against women. In order to instill the importance of women’s career advancement and encourage women to pursue their career ambitions, the GoS and relevant national and international stakeholders should make enshrining codes of conduct, safety and anti-harassment policies at a national level a priority. In addition, the GoS should publicly endorse policies that prohibit discrimination against hiring, promoting and involving women within the workplace. A vocal and staunch advocation against discrimination and for women’s inclusion and career development can help women feel like government policymakers are on their side. Ultimately, policymaking presents an opportunity for the government to lead by example. Therefore, the codification of these policies should be accompanied by Sudan-specific trainings on legacies of exclusion and the future of women’s career advancement, that centers on the societal factors that have acted as barriers to women’s career development.

Lastly, and with relation to leading by example, the GoS and political stakeholders can instill policies that promote the inclusiveness of women’s unions into politics and the representation of women from different social classes at federal level can play an important role in diversifying and heightening women’s voices and concerns at the highest levels of policy change. With regards to Sudan’s current transformative period, quota systems can be instilled within political parties, trade unions, and civil society organizations. As mentioned in the Wilson Centre’s study on women’s public leadership in the MENA region, quotas can support women in gaining political representation with increasing effectiveness over time (Khurma et al., 2020). In addition, increasing women’s representation at these levels can have motivating effects on women throughout Sudan through providing role models for women’s leadership and by demonstrating that women’s demands in society are being discussed within the political sphere. Finally, the government’s support for women in politics would emphasize the important role that women leaders can play as agents of power and change during this critical time in Sudanese politics (Khurma et al., 2020). Public buy-in of women in politics can shape how society views women leaders and encourage more women to engage in this sphere.

## 7. Theoretical implications

This paper also has theoretical implications as follows. This study has demonstrated that career motivation among women in Sudan are shaped by societal factors which prescribe certain characteristics and social roles to women. In turn, this produces cycles of bias, sexism and stereotyping that can impact their career progression. There are no other studies that examine the impact that societal factors play on career motivations of Sudanese women only. The results of this study suggest that an understanding of the societal factors that influence women’s career motivation in Sudan can be utilized by policymakers to support women’s career ambitions and advancement in Sudan.

## 8. Limitations and future research directions

There are two key limitations of this research that should be noted. Firstly, most of the study participants were highly educated women, many with masters and doctorate degrees, and therefore this study is limited in the perspectives of women who do not have a degree in higher education. Therefore, research results can be further tested with a larger more representative sample of women in Sudan from diverse educational backgrounds. Secondly, participants of this study were all identified as women leaders. While this was done to capture women’s motivations and experiences throughout their career journeys, it excludes groups of women who may not identify as leaders as such. Future research can include entry-level professionals or recent graduates, in order to provide a diverse outlook on women’s career motivations in Sudan.

## 9. Conclusion

This is one of the first studies to conceptualize women’s career motivations in Sudan and make significant contributions to understanding the factors that impact them. Through an in-depth analysis of critical literature, empirical findings of a three-round Delphi study, this paper has revealed the personal, psychological, and organizational factors that intersect with societal norms and expectations, which influence Sudanese women’s career motivation and advancement. This paper has demonstrated how psychology and organizational behavior theories, namely implicit bias, stereotype threat and self-efficacy theories, can play a key role in understanding the barriers and enablers that influence women’s career motivations and aspirations. Understanding of these theories and their relevance has resulted in this study offering policy recommendations and implications for key stakeholders interested in driving and supporting women’s career motivations in Sudan. These recommendations are important to lead and foster women’s career progression across sectors and against the backdrop of Sudan’s current political, social, and economic situation, this study is not only timely, but urgent.

While studies on career motivation of Sudanese women remains an understudied area, this study has demonstrated that the experiences of Sudanese women are unique. Therefore, stakeholders hoping to support women’s career motivations in Sudan should develop country-specific solutions that platforms the experiences of Sudanese women across sectors. The findings of this study can be applied to other countries of similar context, particularly where societal norms shape women’s career motivations and experiences. The policy recommendations and implications presented here can also be a starting point to develop gender responsive policies and solutions for countries that are facing similar challenges.

## Data availability statement

The datasets presented in this article are not readily available because of ethical reasons, to protect the participants’ anonymity according to the consent they provided. Requests to access the datasets should be directed to the corresponding author.

## Ethics statement

This study was conducted in accordance with the ethical standards of the London School of Economics and Political Science (LSE) including consent, data protection and confidentiality. All participants of the study were informed of the objectives and procedures prior to partaking in the study and were required to consent forms before the study began. Participants were informed that their participation was voluntary, and all data was treated anonymously. Informed consent forms were completed by all participants and an ethical review on data management were completed and approved by the LSE.

## Author contributions

SM led the research project, designed the methodology, wrote the initial literature overview, and contributed the underpinning psychology and organizational behavior theories in the manuscript and also led the recruitment of the participants as part of a larger study on Women Leadership Development in the MENA region. AA collected, coded, analyzed the data, wrote the initial literature review and the initial drafts of the manuscript and both SM and AA contributed substantially to the manuscripts theoretical development and editing process. HA provided support to both SM and AA throughout and contributed to the recruitment of participants for the study. All authors contributed to the article and approved the submitted version.

## Conflict of interest

The authors declare that the research was conducted in the absence of any commercial or financial relationships that could be construed as a potential conflict of interest.

## Publisher’s note

All claims expressed in this article are solely those of the authors and do not necessarily represent those of their affiliated organizations, or those of the publisher, the editors and the reviewers. Any product that may be evaluated in this article, or claim that may be made by its manufacturer, is not guaranteed or endorsed by the publisher.

## References

[ref1] Abalkhail J. M. (2019). Women’s career development in an Arab Middle Eastern context. Hum. Resour. Dev. Int. 22, 177–199. doi: 10.1080/13678868.2018.1499377

[ref2] Alqahtani T. (2021). Women’s leadership in higher education in the Kingdom of Saudi Arabia. J. Entrep. Organ. Manag 10, 1–5.

[ref3] Alzougool B. Almansour J. Alajmi M. (2021). Women’s leadership styles in the public sector in Kuwait: the perspective of their subordinates. Manag. Sci. Lett. 11, 465–472. doi: 10.5267/j.msl.2020.9.021

[ref4] Bandura A. (1977). Self-efficacy: toward a unifying theory of behavioural change. Psychol. Rev. 84, 191–215. doi: 10.1037/0033-295X.84.2.191 847061

[ref5] Chan P. (2022). An empirical study on data validation methods of Delphi and general consensus. Data 7:18. doi: 10.3390/data7020018

[ref6] Cheriyan C. Shevchuk-Hill S. Riccio A. Vincent J. Kapp S. K. Cage E.. (2021). Exploring the career motivations, strengths, and challenges of autistic and non-autistic university students: insights from a participatory study. Front. Psychol. 12:719827. doi: 10.3389/fpsyg.2021.719827, PMID: 34744884 PMC8568013

[ref7] Chikoore C. Hasabo A. (2015). Supporting the role of women leaders in Sudan and South Sudan in the post-separation period. End of Term Evaluation. United Nations Democracy Fund.

[ref8] Clance P. R. Imes S. A. (1978). The imposter phenomenon in high achieving women: dynamics and therapeutic intervention. Psychother. Theory Res. Pract. 15, 241–247. doi: 10.1037/h0086006

[ref9] Davies P. Spencer S. Steele C. (2005). Clearing the air: identity safety moderates the effects of stereotype threat on women’s leadership aspirations. J. Pers. Soc. Psychol. 88, 276–287. doi: 10.1037/0022-3514.88.2.276, PMID: 15841859

[ref10] Edmondson A. C. (1999). Psychological safety and learning behavior in work teams. Adm. Sci. Q. 44, 350–383. doi: 10.2307/2666999

[ref11] Edmondson A. C. (2018). The fearless organization: Creating psychological safety in the workplace for learning, innovation, and growth. Hoboken, NJ: John Wiley & Sons.

[ref12] Etang A. Lundvall J. Osman E. Wistrand J. (2021). Towards a more inclusive economy: understanding the barriers Sudanese women and youth face in accessing employment opportunities. World Bank. Available at: https://openknowledge.worldbank.org/handle/10986/36104 (Accessed August 29, 2022).

[ref13] Evans K. Maley J. (2020). Barriers to women in senior leadership: how unconscious bias is holding back Australia’s economy. Asia Pac. J. Hum. 59, 204–226. doi: 10.1111/1744-7941.12262

[ref14] Fragile State’s Index (2020). In the wake of Al-Bashir, women of Sudan bring a glimmer of Hope. Available at: https://fragilestatesindex.org/2020/05/10/women-of-sudan-bring-a-glimmer-of-hope/ (Accessed August 28, 2022).

[ref15] Gikandi H. (2021). After the revolution Sudanese women ask, what’s next? The World. Available at: https://theworld.org/stories/2021-08-06/after-revolution-sudanese-women-ask-what-s-next (Accessed July 12, 2022).

[ref16] Global Gender Gap Report (2020). Reports – world economic forum. Available at: https://reports.weforum.org/global-gender-gap-report-2020/ (Accessed July 12, 2022).

[ref17] Greenwald A. G. Banaji M. R. (1995). Implicit social cognition: attitudes, self-esteem, and stereotypes. Psychol. Rev. 102, 4–27. doi: 10.1037/0033-295X.102.1.4, PMID: 7878162

[ref18] Guettaoui A. Hadja O. (2021). Women’s participation in political life in the Arab state. Alg. Sci. J. Platform 4, 93–105. doi: 10.29039/02061-6-93-105

[ref19] Halim A. A. (2019). A woman’s place: can international law assist in removing gender disparities in Sudanese laws? Ahfad J. 36, 33–48.

[ref20] Hameli A. Metzanis C. Kampouris I. (2023). Women’s empowerment conditions, institutions and firm performance in the MENA region. Account. Forum. 1–30. doi: 10.1080/01559982.2023.2179866

[ref21] Hoare J. Gell F. (2009). Women’s leadership and participation: case studies on learning for action. Rugby, Practical Action Publishing. p. 1–19.

[ref01] Hutchings K. Weir D. (2006). Guanxi and wasta: a comparison. Thunderbird International Review 48, 141–156. doi: 10.1002/tie.20090

[ref23] Indexmundi (2019). Sudan – Labour force participation rate, female (% of female population ages 15+) (modelled ILO estimate). Available at: https://www.indexmundi.com/facts/sudan/indicator/SL.TLF.CACT.FE.ZS (Accessed January 23, 2022).

[ref24] International Labour Organization (2020). Women in managerial and leadership positions in the G20 – Data availability and preliminary findings. Geneva: International Labour Office.

[ref26] Kemp L. J. (2012). Progress in female education and employment in the United Arab Emirates towards millennium development goal (3) – gender equality. Foresight J. Future Stud. Strat. Thinking Policy 15, 264–277. doi: 10.1108/FS-02-2012-0007

[ref27] Khurma M. Pagan C. Farley A. Horner A. (2020). Ready to lead: understanding women’s public leadership in the Middle East and North Africa. Wilson Centre, pp. 7–35.

[ref28] Kim M. S. (2020). Concept mapping of career motivation of women with higher education. Front. Psychol. 11:1073. doi: 10.3389/fpsyg.2020.01073 32536894 PMC7269102

[ref29] Kondracki N. L. Wellman N. S. Amundson D. R. (2002). Content analysis: review of methods and their applications in nutrition education. J. Nutrit. Educat. Behav. 34, 224–230. doi: 10.1016/S1499-4046(06)60097-3 12217266

[ref30] Laffineur C. Tavakoli M. Fayolle A. Amara N. Carco M. (2018). “Insights from female entrepreneurs in MENA countries: barriers and success factors” in Entrepreneurship Ecosystem in the Middle East and North Africa. eds. Faghih N. Zali M. R. (Cham: Springer International Publishing), 351–397.

[ref31] Laffrey S. Page G. (1989). Primary health care in public nursing. J. Adv. Nurs. 14, 1044–1050. doi: 10.1111/j.1365-2648.1989.tb01516.x 2613958

[ref32] Linstone H. A. Turoff M. (2002). Delphi method: techniques and applications. Addison-Wesley Publishing Company, Reading, MA.

[ref33] Mahmoud F. (2005). Democratic transformation and the participation of women in political institutions in the Sudan. Respect 1st issue, London.

[ref34] Marshall M. (1996). Sampling for qualitative research. Fam. Pract. 13, 522–526. doi: 10.1093/fampra/13.6.522 9023528

[ref35] Maslow A. H. (1943). A theory of human motivation. Psychol. Rev. 50, 370–396. doi: 10.1037/h0054346

[ref37] Mohamed S. Abbashar A. (2022). Investigating women’s leadership development in Sudan. Middle East Centre, London School of Economics and Political Science Blogs Series. Available at: https://blogs.lse.ac.uk/mec/2022/08/18/investigating-the-barriers-and-enablers-for-womens-leadership-in-sudan/ (Accessed January 22, 2023).

[ref38] Osman A. (2014). Beyond the pan-Africanist agenda: Sudanese women’s movement, achievements and challenges. Fem. Africa 19, 43–55.

[ref39] Palinkas L. Horwitz S. Green C. Wisdom J. Duan N. Hoagwood K. (2015). Purposeful sampling for qualitative data collection and analysis in mixed method implementation research. Adm. Policy Ment. Health 42, 533–544. doi: 10.1007/s10488-013-0528-y, PMID: 24193818 PMC4012002

[ref40] Patel P. Meagher K. El Achi N. Gemma G. Ekzayez A. Suvllivan R. (2020). Having more women humanitarian leaders will help transform the humanitarian system’: challenges and opportunities for women leaders in conflict and humanitarian health. Confl. Heal. 14:84. doi: 10.1186/s13031-020-00330-9, PMID: 33292351 PMC7709302

[ref41] Perkins D. N. (1992). Smart schools: better thinking and learning for every child. New York: Free Press.

[ref42] Pillay-Naidoo D. Nel P. (2022). Testing a model of resilience for women leaders: a strengths-based approach. Aust. J. Psychol. 74:2138542. doi: 10.1080/00049530.2022.2138542

[ref43] Rivi A. (2022). MENA ‘needs more female leaders to close 115-year gender gap.’ The National News. Available at: https://www.thenationalnews.com/uae/government/2022/07/13/mena-region-needs-more-female-leaders-to-close-115-year-gender-gap-global-report-finds/

[ref44] Schock A. K. Gruber F. M. Scherndl T. Ortner T. M. (2019). Tempering agency with communion increases women’s leadership emergence in all-women groups: evidence for role congruity theory in a field setting. Leadersh. Q. 30, 189–198. doi: 10.1016/j.leaqua.2018.08.003

[ref45] Serfaz A. Mehta M. A. (2019). Women leadership and organizational barriers: a socio-economic and ethical point of view. J. Leg. Ethical Regul. Issues 23, 1–9.

[ref47] Steele C. M. Aronson J. (1995). Stereotype threat and the intellectual test performance of African Americans. J. Pers. Soc. Psychol. 69, 797–811. doi: 10.1037/0022-3514.69.5.797, PMID: 7473032

[ref48] Storberg-Walker J. Madsen S. R. (2017). The women and leadership theory think report. George Washington University. Available at: https://www.usu.edu/uwlp/files/wlthinktankreport2015.pdf (Accessed January 22, 2023).

[ref49] The World Bank (2019). Women, business and the law: a decade of reform, Washington, DC: The World Bank Report.

[ref50] Tlaiss H. Kauser S. (2010). Perceived organizational barriers to women’s career advancement in Lebanon. Gend. Manag. Int. J. 25, 462–496. doi: 10.1108/17542411011069882

[ref51] Tonnnessen L. Al-Naggar S. (2020). Number 2: Sudan brief June 2020 – patriarchy, Politics and women’s activism in post-revolution Sudan. CHR Michelsen Institute Report.

[ref52] UN Women (2017). Fund for gender equality 2016 annual report. UN Women Report.

[ref53] UN Women (2019). From Sudan to the Security Council: Sudanese women lead drive for change. Available at: https://www.unwomen.org/en/news/stories/2019/11/feature-sudanese-women-lead-drive-for-change. (Accessed April 24, 2023).

[ref54] UNDP (2020). Finding Sudan’s future women political leaders: 1070 and rising. UNDP Sudan. Available at: https://sudan.un.org/en/49556-finding-sudans-future-women-political-leaders-1070-and-rising (Accessed July 12, 2022 and April 24, 2023).

[ref55] UNICEF (2021). Gender Annual Report 2021. UNICEF Sudan Report.

[ref56] Young S. (2020). The women’s revolution: female activism in Sudan. Harv. Int. Rev. Available at: https://hir.harvard.edu/the-womens-revolution-female-activism-in-sudan/ (Accessed January 28, 2023).

